# COVID-19 pandemic: a catalyst for transformation of a summer online research program

**DOI:** 10.1080/10872981.2021.1886029

**Published:** 2021-02-10

**Authors:** Behnoosh Afghani

**Affiliations:** Department of Pediatrics, University of California Irvine, Orange, CA, USA

**Keywords:** COVID-19, online research, summer research, innovative research program, remote research, evidence-based research

## Abstract

In view of limited resources during the COVID-19 pandemic, there is an urgent need to create novel programs to meet the changing demands of trainees towards developing and strengthening their skills in healthcare research. During the COVID-19 pandemic, digital learning has become an invaluable tool by providing more learning opportunities. Through the use of platforms available for distant learning, we made our pre-existing online research program more interactive. Through collaboration in small groups, the trainees developed their research and mentorship skills and were able to meet the goal of submitting their research projects as abstracts. All of the abstracts were accepted for publication.

## Education challenge

Because of the COVID-19 crisis, many trainees have paused their research and are seeking opportunities to restart their research efforts and strengthen their skills while others have had to modify their projects. There is a major concern that an increasing number of trainees who are seeking competitive post-graduate training or fellowships are pressured to take an extra year of research to make up for the lost opportunities [1]. Therefore, there is an urgent need to develop remote programs that would help college and medical students participations in research and stimulate their interest in health challenges that are currently afflicting people worldwide.

## Substance of innovation

We describe an innovative Online Research Program developed at the University of California, Irvine that trains premedical and medical students about the basics of healthcare research with an emphasis on literature review and evidence-based medicine. The cascading nature of our program has been described previously [2]. Under the direction of a faculty mentor, college and medical student coaches guide small groups of high school students to complete a research project during a three-week period. The program is sustainable as 80% of high school students pay tuition to participate, and the rest receive scholarships. Between 2015 and 2019, all of the instruction was done through pre-recorded podcasts and sharing of google documents. As a result of the COVID-19 pandemic, we not only made our program more engaging and interactive by using Zoom virtual web-based communication platform (Table 1), we also stimulated the interest of students on topics related to COVID-19 and health disparities. Several of the coaches had participated in our other pipeline programs as a coach or high school student previously. During the summer of 2020, two 3-week sessions were held under the direction of one faculty mentor (author BA). There was a total of 52 students, 26 per session, divided into 5 groups of 5–6 students per group. The groups in each session completed a different project under the mentorship of a different coach with five coaches per session. The students worked in teams and learned about the main components of a research project, such as designing, conducting, and finalizing a project as well as preparing an abstract. The students in each group were expected to collaborate through shared google documents on the different sections of their unique project: Background, Methods, Results, and Discussion. Other students in the same group as well as the coach and the faculty mentor reviewed and provided feedback on the student’s work. Through collaboration, the students were able to complete the project by the given deadline and presented their findings to the whole group in an oral presentation at the end of the session. After the program ended, the faculty mentor worked with each coach to prepare an abstract for publication.

**Table 1.** Comparison of the previous (Pre-COVID-19) and current model (COVID-19) programs

Session	Pre COVID-19 Summer	During COVID-19 summer
Introduction to evidence based medicine	Pre-recorded by the PD*	Virtual session by PD
How to do a literature search	Pre-recorded by the PD	Virtual session by PD
Patient Privacy Talk	Web-based learning	Virtual session by PD
Explanation of each group’s topic	Pre-recorded by PD	Virtual session by each coach
Each team working on the project	Sharing through google docs	Sharing through google docs
Questions and Answers	Through email	Virtual office hours, multiple times
College and career advice by the coaches	None	Virtual session by each coach
End of the program presentation	None	Virtual presentation of each project by the students on the last day of the program
Activities after program completion	PD guides the coaches in person or via phone to assemble the final abstract	PD guides the coaches virtually to assemble the final abstract

*PD = Program Director (faculty mentor)

In this study, we only focus on the feedback from the coach mentors. All of the coaches responded to our survey. Based on a 4-point Likert Scale ranging from 1 = decreased and 4 = significantly increased, the average rating of the coaches on the impact of the program in promoting their interest in research was 3.6, their leadership skill was 3.9, their mentorship skills were 3.9, and their ability to do a literature review was 3.7. All of the coaches rated their satisfaction with the structure of the program and their research topic at the highest rating of 4. The qualitative feedback survey included the following comments:

- *I am really glad that I was able to participate in this program and develop my research skills and my mentoring capabilities.*
- *The program was very beneficial for both the coaches and students! The faculty mentor is very responsive to any questions/concerns throughout the program. I also appreciated the flexibility with a schedule that this program allowed. Overall, a wonderful experience that I would love to participate in again.*
- *Overall, I think the second time around I felt more confident in organizing my time and communicating with the rest of the students. I also feel like I gleaned a lot of information from the faculty mentor about residency and fellowship programs and the importance of work–life balance.*
- *I completed this program with the same faculty mentor last year and I really enjoyed it. I like that there were zoom meetings and more interaction this year in comparison to last year-a great improvement.*

Our goal was met as all of the 10 abstracts submitted by the coaches as the first author were accepted for publication in the Journal of Investigative Medicine. Of the 10 abstracts, five were related to health inequalities and the other 5 were related to cutting-edge COVID-19 research topics.

## Lessons learned

We believe that there were several reasons for the effectiveness of our program during the summer of 2020. In their feedback, the returning coaches stated that they preferred the 2020 program compared to the previous years because the interactive and collaborative nature of live video meetings in real time helped them to be innovative and develop their mentorship and leadership skills. As Palloff describes, collaborative learning that allows sharing of ideas among the students is very important in forming the foundation of a learning community [3]. The coaches and students found the flexibility of attending the program around other work and school responsibilities as an advantage. Students from different areas in the USA participated in our programs, and although students were not all from the same time zones, the small group design allowed the coaches and students to decide on meeting times that were convenient for all of the students in the group. As previously described [2], there were three components in the cascading nature of our Online Research Program. Under the supervision of one expert faculty mentor, the college or medical student mentors (also known as coaches) guided the high school students to complete their research projects through frequent virtual meetings and formal presentations. In addition, the coaches provided college and career development advice to the mentees throughout the program and after the session ended. Prior to the sessions, the faculty expert met with the coaches to determine a topic of interest unique to each coach, and she provided continuous support and feedback throughout the program. Near-peer mentoring of mentees close in age has been shown to benefit both the mentors and mentees in STEM (Science, Technology, Engineering, and Math) fields [4]. The less experienced mentees benefit from the mentor’s knowledge and skills, and the mentors develop their teaching and leadership skills as they guide the mentees [5]. In models that include faculty experts, the faculty mentors encourage the college level mentors to perceive the mentees' progress as a personal responsibility [2,4].

Finally, faculty mentor’s commitment to mentorship contributed to the success of the program. She encouraged a comfortable but goal-oriented environment where the coaches provided guidance to students through near-peer mentorship. Having known a few of the coaches over the years, the faculty mentor was able to assess each of their capabilities and develop an individualized plan to make sure the coach would succeed in mentoring high school students and completing their research projects with the goal of publication. The goal of publishing all projects as abstracts as well as the positive responses from the coaches suggest that our virtual-based research program has been successful and can be implemented at other institutions.
